# Nuclear factor erythroid 2–related factor 2 (Nrf2) deficiency causes age-dependent progression of female osteoporosis

**DOI:** 10.1186/s12891-022-05942-1

**Published:** 2022-11-25

**Authors:** Yusuke Kubo, Jesus Abraham Herrera Gonzalez, Rainer Beckmann, Marek Weiler, Helda Pahlavani, Mauricio Cruz Saldivar, Katharina Szymanski, Stefanie Rosenhain, Athanassios Fragoulis, Sander Leeflang, Alexander Slowik, Felix Gremse, Michael Wolf, Mohammad Javad Mirzaali, Amir Abbas Zadpoor, Christoph Jan Wruck, Thomas Pufe, Mersedeh Tohidnezhad, Holger Jahr

**Affiliations:** 1 Department of Anatomy and Cell Biology, Uniklinik RWTH Aachen, Wendlingweg 2, 52074 Aachen, Germany; 2 Institute for Experimental Molecular Imaging, Helmholtz Institute for Biomedical Engineering, Uniklinik RWTH Aachen, Forckenbeckstraße 55, 52074 Aachen, Germany; 3 Department of Biomechanical Engineering, Faculty of Mechanical, Maritime, and Materials Engineering, Delft University of Technology (TU Delft), Mekelweg 2, 2628 CD Delft, The Netherlands; 4 Department of Orthodontics, Uniklinik RWTH Aachen, Pauwelsstraße 30, 52074 Aachen, Germany; 5 Institute of Structural Mechanics and Lightweight Design, RWTH Aachen University, Wüllnerstraße 7, 52062 Aachen, Germany

**Keywords:** Nrf2, Fracture resilience, Bone strength, Osteoporosis, Estrogen

## Abstract

**Background:**

Nuclear factor erythroid 2–related factor 2 (Nrf2) is a crucial transcription factor for cellular redox homeostasis. The association of Nrf2 with elderly female osteoporotic has yet to be fully described. The aim was to elucidate a potential age-dependent Nrf2 contribution to female osteoporosis in mice.

**Methods:**

Eighteen female wild type (WT) and 16 Nrf2-knockout (KO) mice were sacrificed at different ages (12 weeks = young mature adult and 90 weeks = old) to analyze their femurs. The morphological properties (trabecular and cortical) were evaluated by micro-computed tomography (μCT) and compared to gold standard histochemistry analysis. The quasi-static compression tests were performed to calculate the mechanical properties of bones. Additionally, the population of bone resorbing cells and aromatase expression by osteocytes was immunohistochemically evaluated and empty osteocyte lacunae was counted in cortical bone.

**Results:**

Old Nrf2-KO mice revealed a significantly reduced trabecular bone mineral density (BMD), cortical thickness, cortical area, and bone fraction compared to old WT mice, regardless of no significant difference in skeletally mature young adult mice between WT and KO. Specifically, while all old WT mice showed thin metaphyseal trabeculae, trabecular bone was completely absent in 60% of old KO mice. Additionally, old KO mice showed significantly more osteoclast-like cells and fewer aromatase-positive osteocytes than WT mice, whereas the occurrence of empty osteocyte lacunae did not differ between both groups. Nrf2-KO mice further showed an age-dependently reduced fracture resilience compared to age-matched WT mice.

**Conclusion:**

Our results suggest that chronic Nrf2 loss can lead to age-dependent progression of female osteoporosis.

## Summary

The association between Nrf2 and osteoporosis has not been elucidated. 90-week-old Nrf2-KO female mice showed reduced trabecular/cortical parameters on micro-CT, reduced peak force to induce fractures in mechanical tests, increased osteoclast-like cells, and decreased estrogen synthase expression, compared to age-matched WT mice. These suggest Nrf2 deficiency may result in osteoporosis.

## Background

Oxidative stress is defined as the consequence of an imbalance between production of oxidants and electrophiles/their elimination by protective mechanisms such as antioxidants [1]. It generally affects bone turnover through reducing osteoblastogenesis but increasing osteoclastogenesis, leading to a net decrease in bone mass, a condition known as osteoporosis [2, 3]. Additionally, intrinsic oxidative stress can cause delayed fracture healing or nonunion fractures due to the irreversible cell damage by excessive toxic radicals [4, 5].

A complex cellular defense mechanism protects cells against exposure to toxic radicals by activating an antioxidant response reaction upon sensing oxidative stress [6]. In this system, nuclear factor erythroid 2–related factor 2 (Nrf2) has been identified as a key transcriptional factor that regulates the expression of antioxidant and detoxifying enzymes. Lippross et al. were the first to show that Nrf2 deficiency impairs bone fracture healing in mice [7], while they were unable to elucidate the biological reason. A recent study now reported that female Nrf2-knockout (KO) mice have a delayed rate of bone acquisition as compared to males [8], which suggests sex steroids are influencing the Nrf2 system in bone.

In other tissues, a link between estrogen and Nrf2 is well established [9–11]; 17β-estradiol can increase Nrf2 to regulate the expression of antioxidant enzymes in the myocardium and exercise-dependent epigenetic de-repression of Nrf2 appeared to be osteoprotective. Estrogen deficiency is further closely related to primary osteoporosis in postmenopausal females. As this hormone inhibits bone resorption, a postmenopausal drop in estrogen levels flips the bone turnover balance in favor of bone resorption [12]. However, the influence of long-term Nrf2 deficiency on the female skeleton in the natural course of aging has yet to be fully described. Improved knowledge of a contribution of Nrf2 and oxidative stress to female osteoporosis would benefit future therapeutic strategies to treat osteoporosis and osteoporotic fractures.

Therefore, we investigated the effects of systemic Nrf2 deficiency on bone structure and biomechanics in aging females using a murine Nrf2 KO model.

## Methods

### Mice

The experimental protocol involving animals was approved by a institutional ethics committee of RWTH Aachen University hospital. Female Nrf2-KO (Nrf2^−/−^) mice on a C57BL/J6 background were generated as described by Fragoulis et al. [13], and female wild-type (WT) (Nrf2^+/+^) mice, littermates of the Nrf2-KO mice, were used as controls. WT and KO mice were randomly assigned to either the young mature adult group (12-week-old) or the old group (90-week-old). In total 34 bone femurs including 18 young adult mice (WT, *n* = 11; KO, *n* = 7), and 16 old mice (WT, *n* = 12; KO, *n* = 4) were used. All animals in this study were centrally housed in the laboratory animal facility of Uniklinik RWTH Aachen (Aachen, Germany) under specific pathogen-free conditions. Up to five animals were caged on a 12 h light/dark cycle with free access to food and water. Once the animals reached the desired age (group 1: 12 weeks; group 2: 90 weeks), they were sacrificed by cervical dislocation in accordance with the German Animal Protection Act (TierSchG), in agreement with regulations of the European Animal Research Association (killing notification protocol No. 40108A4). Dissection of the hind limbs was performed postmortem, and frozen at − 80 degree.

### Micro computed tomography (μCT) device

Extracted murine whole femurs were scanned using a Bruker SkyScan 1272 μCT system (Bruker, Belgium) [14, 15]. The Bones were taken out of the freezer and immediately put into the sample tube and kept in a wet condition by humid tissues at the top and bottom of the tube. Before scanning the samples, a 3 h-preheating phase was done to adjust to the scanner temperature. The artifacts were prevented by these procedures. The x-ray source was operated at 65 kV and 0.13 mA with a 0.25 mm aluminum attenuation filter. Projections (1344 × 896 pixels) were acquired in a circular trajectory and used to reconstruct a volume with isotropic voxel size 13-μm. We used an exposure time of 65 ms and a rotation step of 0.4° and frame averaging of 6 in the anteroposterior axis. Acquisition time for each specimen was 150 minutes and batch acquisition was performed with up to 3 specimens suspended in a single sample tube. Scan analysis was carried out using the software ‘Imalytics Preclinical’ (Gremse IT GmbH) [16].

### Trabecular bone parameters

**Fig. 1:**
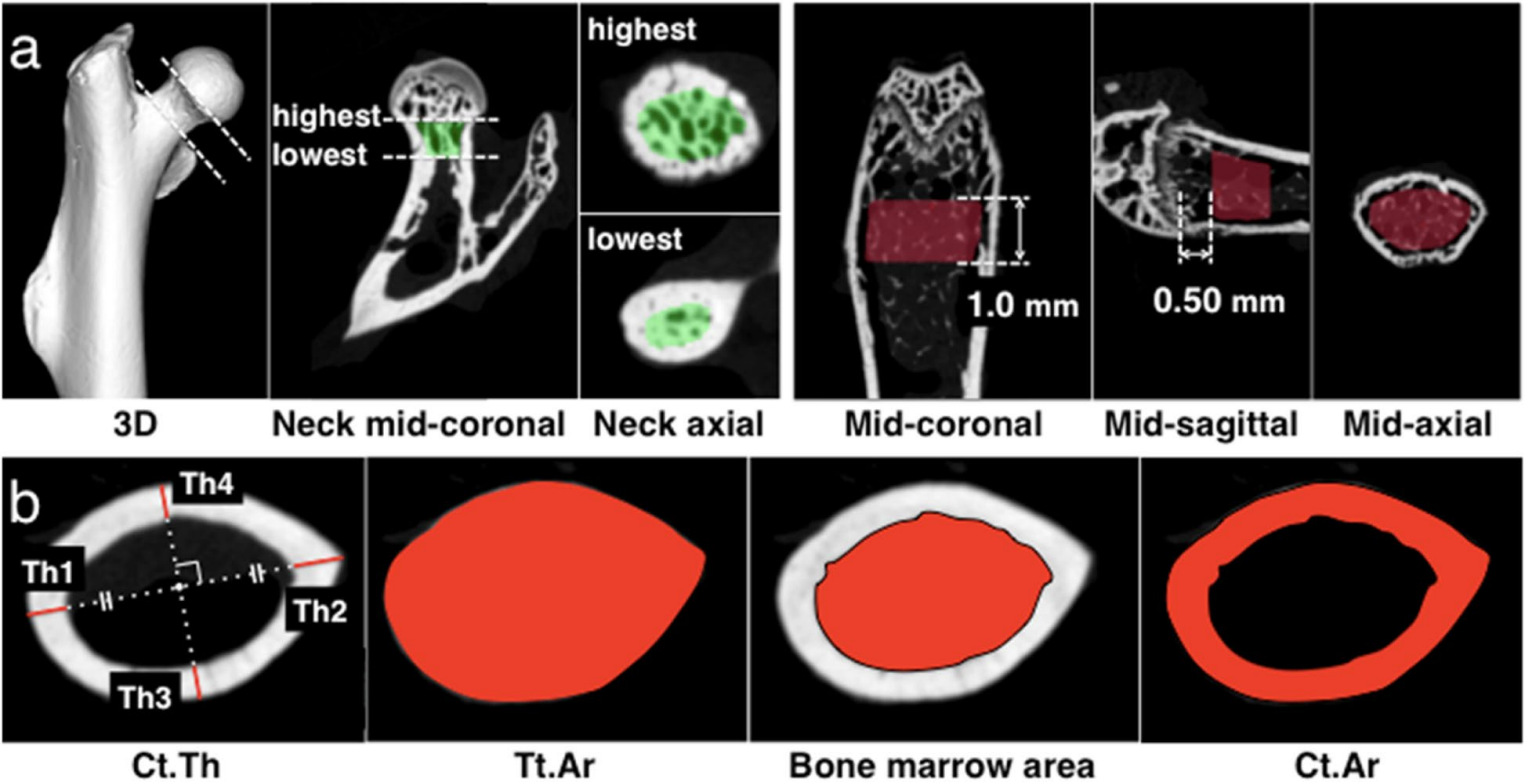
Trabecular and cortical bone parameters by μCT The trabecular bone parameters in the femoral neck were measured in the range (green region) of from femoral head-neck junction (highest) to the junction with the greater trochanter in the axial view of the neck (lowest). On sagittal view of distal femoral metaphysis, measurement area was 0.50 mm far from the growth plate. A region of thickness of 1.0 mm was placed covering the trabecular bone and excluding cortical bone **(a)**. On axial view of femoral neck and midshaft, cortical thickness (Ct.Th) was defined as the mean value of cortical thicknesses on major axis (Th1 and Th2) and the ones on perpendicular bisector of major axis (Th3 and Th4). Ct. Th (μm) was expressed as (Th1 + Th2 + Th3 + Th4) / 4 (μm). The analysis ranges of total cross-sectional area (Tt.Ar) (mm^2^). Cortical bone area (Ct.Ar) (mm^2^) was obtained by substracting bone marrow area from Tt. Ar **(b)**. CT, computed tomography

Trabecular bone parameters were measured in the femoral neck and distal metaphysis. The femoral neck area was determined by the range from femoral head-neck junction to the junction with the greater trochanter in the axial view of the neck [17]. Distal femoral metaphysis, most commonly assessed in mice [18], was evaluated as a suitable trabecular bone parameter. The scans of the femur typically start proximal to the growth plate to capture the metaphyseal region [18]. Measurement range was decided at a distance of 0.50 mm from the growth plate to avoid the effects of the growth plate [18, 19]. The range for analysis in the axial direction was 1.0 mm [18]. Regarding the selection of the region of interest (ROI), contours were drawn manually in each slice to separate the trabecular bone from the cortical wall based on the previous report [20] (Fig. 1 a). As trabecular bone parameters, trabecular thickness (Tb.Th) (μm), the mean thickness of trabeculae assessed using direct 3D methods; bone mineral density (BMD) (IU); bone surface (BS) (mm^2^), the surface of the region segmented as bone; and bone volume fraction (BV/TV) (%), the ratio of the segmented bone volume to the total volume of the region of interest, were computed [14].

### Cortical bone parameters

Cortical bone parameters were measured at the axial region of femoral neck and midshaft according to the typical procedure described by previous report [17, 18]. The analysis region of femoral neck was mid-axial of femoral neck, and that of femur was centered on the midpoint of the long bone, determined as half the distance from the distal condyle to the proximal point of the femoral head on μCT [21]. As cortical bone parameters, cortical thickness (Ct.Th) (μm), total cross-sectional area (Tt.Ar) (mm^2^), cortical bone area (Ct.Ar) (mm^2^), and cortical bone fraction (Ct.Ar/Tt.Ar) (%) were determined [14]. All these parameters were manually measured using the ImageJ software (National Institutes of Health, Bethesda, MD, USA), a free open-source image-processing platform that provides extensibility via Java plugins and recordable macros (Fig. 1 b).

### Settings of analysis parameters

The μCT images were binarized for the analysis of bone structure by setting a grey-level threshold, above which voxels are considered bone and below which voxels are considered background. Although the ideal threshold is the midpoint between the background and bone peaks in the histogram when selecting the threshold visually, the choice of threshold can vary from user to user [22]. Therefore, a fixed threshold of 10,000, a raw grey scale corresponding to the provided values in the TIFF-files (range, 0 to 65,535) was determined to show reasonable binarization of bone structure based on the previous paper and kept the same threshold for the entire study [14].

### Histopathology

The resected murine femurs were fixed overnight in methacarn solution, including 60% absolute methanol, 30% chloroform, and 10% glacial acetic acid - freshly prepared before fixation. Subsequently, the samples were decalcified in a 10% ethylenediaminetetraacetic acid (EDTA) buffer for 2 weeks. Afterwards, the samples were washed under running tap water, soaked in 70% ethanol and embedded in paraffin. Then, histopathological specimens were stained with hematoxylin and eosin (HE).

The mean ratio of empty osteocyte lacunae in mid-cortical was calculated in young adult and old WT and Nrf2-KO mice. The slides were imaged using Keyence BZ-9000 microscope (Keyence, Osaka, Japan). We randomly set at least five equally ROIs (65μm^2^) in each mid-diaphysis of slices. The mean number was then calculated for all values per section. We used three sections in each specimen. The mean number of osteoclasts per mm^2^ in the distal femur was calculated in three mid-sagittal slices of each histological specimens. Among several kinds of multinuclear giant cells, osteoclasts can be characteristically identified as multinuclear cells along with or within the indentations of the bone matrix [23, 24]. The number of these osteoclast-like cells in the distal femur area was counted and compared between WT and KO mice in young adult and old age.

Aromatase-positive osteocytes in femur mid-diaphysis were evaluated in young adult and old WT and KO mice. Paraffin-embedded tissue sections were deparaffinized with xylene and alcohol to distilled water. The sections were heated with citrate buffer (pH = 6.0), blocked with 3% hydrogen peroxide and washed with phosphate buffer saline (PBS), and serum block was performed with 3% normal horse serum for antigen retrieval. Immunohistochemistry was performed using rabbit primary antibody against aromatase (1:100 in antibody diluent; BioVision: Cat#3599–30 T), and the tissue sections were incubated at room temperature for 1 h. After washed with PBS, this procedure was followed by horse anti-rabbit secondary antibody for aromatase, and then washed with PBS. Counterstaining was performed with hematoxylin staining. The numbers of aromatase-positive osteocytes and total cells were counted. Subsequently, aromatase-positive osteocyte counts per 1 mm^2^ as well as their ratios (out of total cell counts) were compared.

### Mechanical loading test and finite element (FE) analysis

The mechanical test was performed on the extracted femurs from young adult and old mice of WT and KO group types. These femurs were contralateral side of the ones used for histological evaluation, and they were frozen at − 80 degree before mechanical testing.

We used a mechanical testing machine (LLOYD instrument LR5K, load cell = 5000 N) to perform a quasi-static compression test (stroke rate = 1 mm/min) under displacement-control conditions. In order to provide a smooth force transmission between the gripping systems of the mechanical test benchmark and femur bones, we embedded the proximal and distal epiphysis parts of the femoral bones in universal acrylic could mounting resin (ClaroCit, Struers Ap5, Ballerup, Denmark). The applied displacement and the reaction force were recorded to obtain the force-displacement curve.

In order to compensate for the bone shaft misalignment, using a high-resolution camera, we took two images in two perpendicular planes from each specimen before mechanical testing. These images were used to calculate the angle to which the recorded force was transformed. We used trigonometric relations for the force transformation. From the mechanical tests, we calculated the stiffness as the slope of the force-displacement curve and the maximum transformed force (Force_max_).

We obtained full-field strain maps (main principal true strains) of two selected tests (one from WT and one from KO group) by using a Q-400 2x 12MPixel Digital Image Correlation (DIC) system (LIMESS GmbH, Krefeld, Germany) and its associated software (Instra 4D v4.6, Danted Dynamics A/S, Skovunde, Denmark) at a frame rate 4 Hz of frequency. We applied a black dot speckle pattern over a white paint background on these specimens to obtain the proper strain values. After processing the images, the software computed the displacements and strains using the deformations within the speckle pattern.

We created voxel-based computational models of representative specimens from 4 different bone groups (i.e.*,* young adult WT and KO mice, and old WT and KO mice) and performed finite element (FE) analysis using a commercial nonlinear solver (Abaqus Standard 2021). The selection criteria was based on choosing specimens with closer stiffness and Force_max_ values to those of average values of their representative groups. To build our computational models, the geometry of each specimen was first reconstructed from the μCT images using an image processing software (Mimics 21.0, Materialise, Leuven, Belgium). The segmented part was then imported into a mesh generating software (3-matic 15.0, Materialise, Leuven, Belgium) to generate surface and volume mesh (element type: C3D4, a 4-node linear tetrahedron solid element). To combine the information of the DICOM images and the volume mesh, the generated volume mesh was imported again into the Mimics software, where the original DICOM files were open. Then, the elastic material properties (i.e.*,* elastic moduli and Poisson’s ratio) were heterogeneously assigned to each voxel of the μCT images based on the relation between the Hounsfield Unit (HU) and the density of the bone. We used a gray value-based method for material property assignment. We defined two material types. The first material type was assigned to all negative HU values until 100 HU and was used to set a lower limit for density (50 kg/m^3^) and avoid negative density values. The second material type was used to cover the range of trabecular and cortical bones (HU > 100) and was assumed to include ten materials with density values ranging from 50 kg/m^3^ to 1700 kg/m^3^. For both material types, we assumed Poisson’s ratio equal to 0.3, and we calculated Young’s modulus based on an empirical equation expressed for the femur (*E* = 0.004*ρ*^2.01^ (MPa)) [25]. We applied a uniform pressure (total force = 10 N) on the femoral head and displacement boundary conditions at the bottom of the femur fixing the bottom of the bone in all directions. For all simulations, we used a static general analysis, and the non-linear geometry option was turned on (NLGEOM = ON).

### Statistical analysis

The μCT, histopathological parameters, mechanical properties were compared between the young adult and old groups and between WT and Nrf2-KO mice, using the Wilcoxon rank sum test. The evaluation of μCT and histopathology was blindly conducted by independent two observers. Values were expressed as means ± standard error of the mean (SEM). Statistical significance was set at *ρ* < 0.05. The correlation between max force in the mechanical testing and cortical parameters was evaluated by using the Spearman’s Rank correlation coefficient. All statistical analyses were performed using the JMP Pro 13 software package (SAS Institute, Cary, NC, USA) or GraphPad Prism (version 8.0.0, GraphPad Software, San Diego, CA, USA).

## Results

**Fig. 2:**
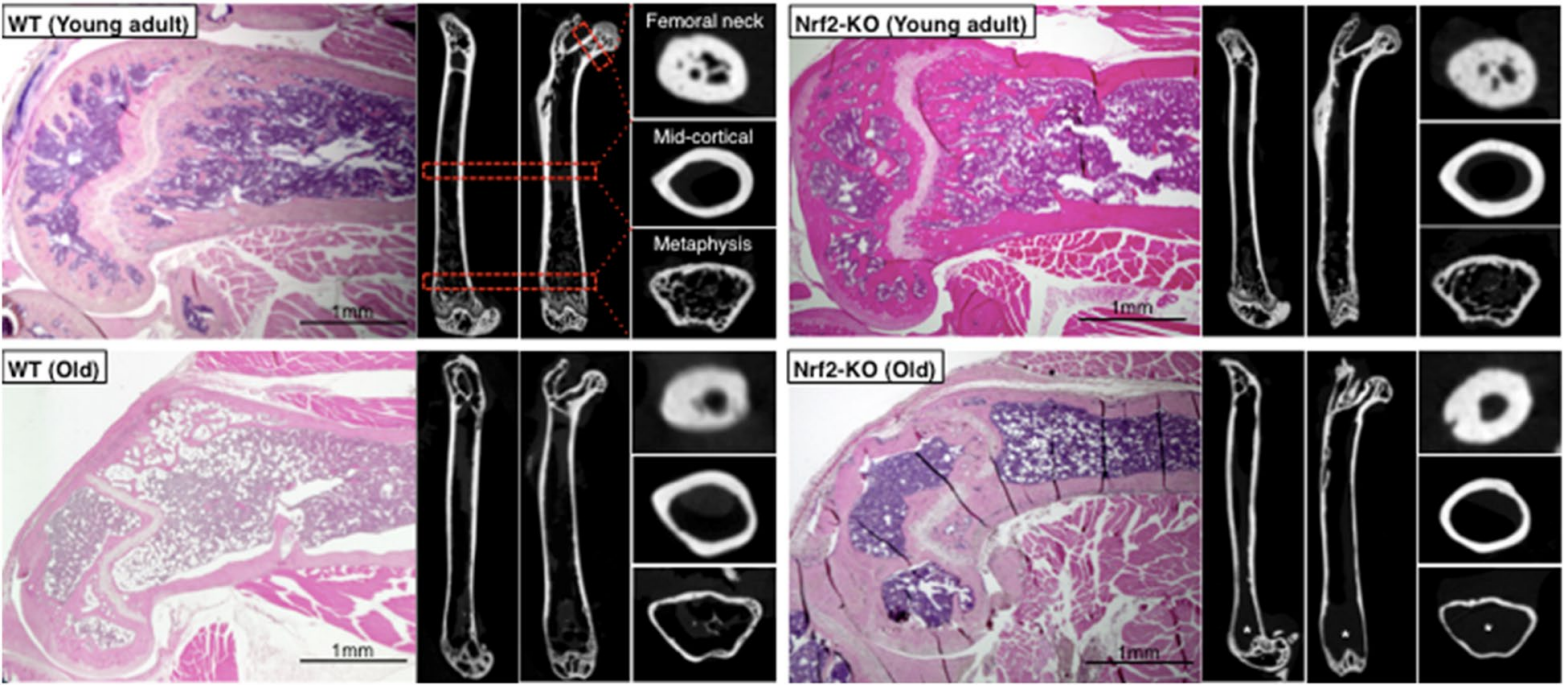
Comparison between histological and μCT images Representative histochemical staining of distal femur (HE, on left) and μCT images of whole femur (sagittal, coronal, and axial planes, respectively) in young mature adult WT and Nrf2-specific KO mice, next to old WT and KO mice. Trabecular bone was absent in old KO mice (asterisks). HE, hematoxylin and eosin; WT, wild-type; Nrf2, Nuclear factor erythroid 2–related factor 2; KO, knockout

The μCT images show abundant metaphyseal trabecular and relatively thick mid-cortical (diaphyseal) bone in distal femurs of young adult WT and Nrf2-specific KO mice in. In contrast, much less trabecular bone and thinner cortical bone was evident in the same anatomical region of old WT mice. In addition to thin cortical bone, no trabecular bone was observed at the distal femur in 60% of old KO mice, while thin trabecular bone was observed in all old WT mice (Fig. 2).

**Fig. 3:**
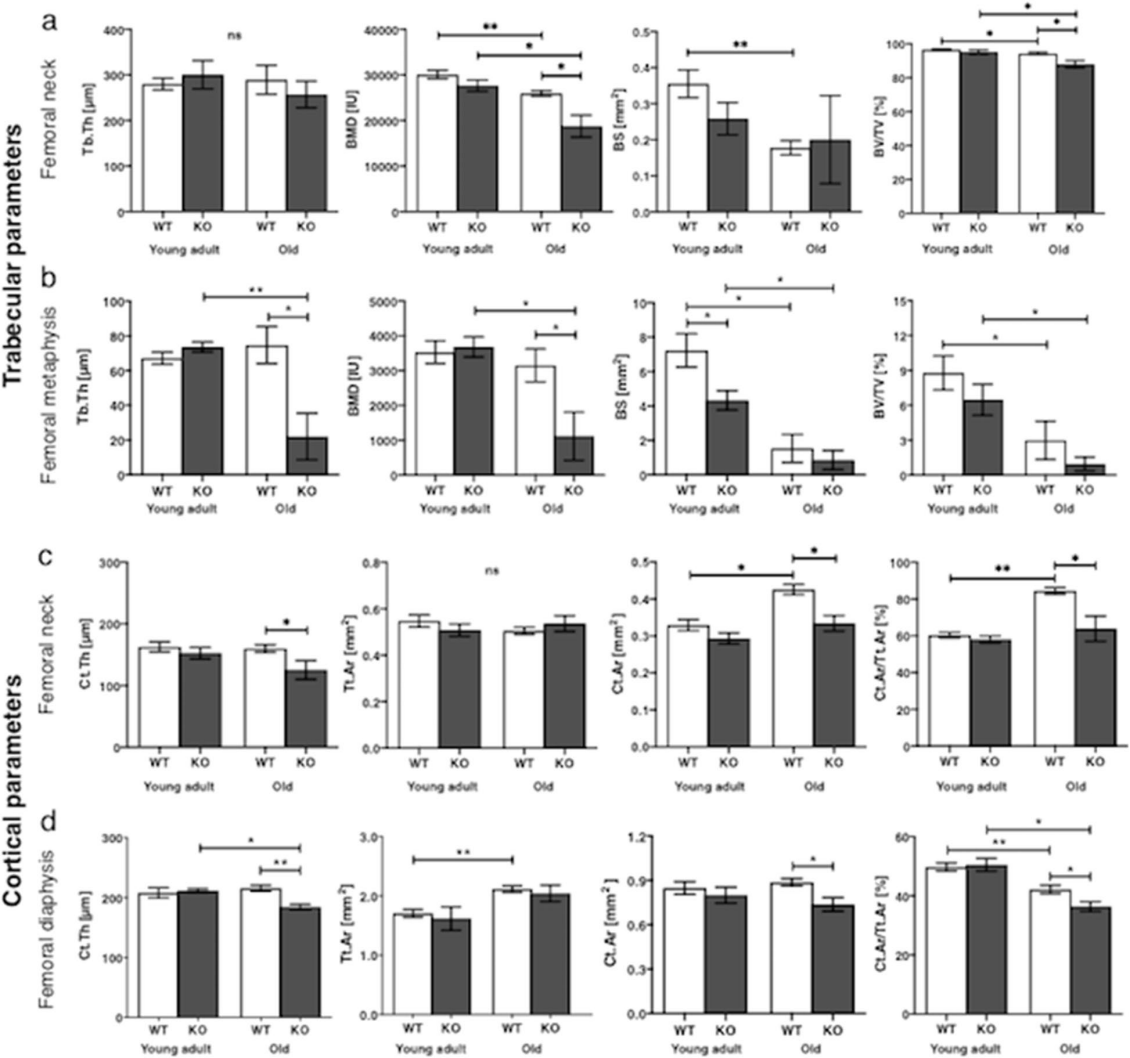
Trabecular and cortical parameters calculated for different bone groups Comparison trabecular (Tb) bone parameters in femoral neck (**a**) and metaphysis (**b**) including Tb. Th (μm), BMD (IU), bone surface (mm^2^), and BV/TV (%) between young adult and old WT and Nrf2- specific KO mice. Comparison of cortical bone parameters of femoral neck (**c**) and diaphysis (**d**) section including Ct. Th (μm), Tt. Ar (mm^2^), Ct. Ar (mm^2^), and Ct.Ar/Tt.Ar (%) between WT and KO mice in young mature and old animals. Wilcoxon rank sum test was used for statistics. Values are means ± SEM. Tb. Th, trabecular thickness; BMD, bone mineral density; BS, bone surface; BV/TV, bone volume fraction; Ct. Th, cortical thickness; Tt. Ar, total cross-sectional area inside the periosteal envelope; Ct. Ar, cortical bone area; Ct.Ar/Tt.Ar, cortical bone fraction; *ρ < 0.05 and **ρ < 0.001 indicate significance; ns, not significant; SEM, standard error of the mean

Femoral neck Tb. Th did not differ between young adult WT and Nrf2-specific KO mice (Fig. 3a). In both, femoral neck and metaphysis, the trabecular BMD of old KO mice was lower than in old WT mice (*p* < 0.05) and young adult KO mice (*p* < 0.05). No significant difference was found in the BS between old WT and KO mice. Young adult KO mice, however, had a smaller BS than young adult WT mice in the femoral metaphysis (*p* < 0.05). Old KO mice showed a reduced BV/TV ratio compared to old WT mice only in femoral neck (p < 0.05) . The BV/TV of old WT and KO mice was lower than that of young adult WT and KO mice, respectively (*p* < 0.05) (Fig. 3a, b).

Old Nrf2-specific KO mice had a reduced Ct. Th compared to old WT mice in femoral neck (*ρ* < 0.05) and diaphysis (p < 0.001), respectively, and also compared to the femoral diaphysis of young adult KO mice (p < 0.05). Tt. Ar did not differ between WT and KO mice. In both femoral neck and diaphysis regions, the Ct. Ar, and Ct.Ar/Tt.Ar of old KO mice was lower than that of old WT mice (*ρ* < 0.05), while no difference in this parameter was seen between young adult WT and KO mice (Fig. 3c, d).

**Fig. 4:**
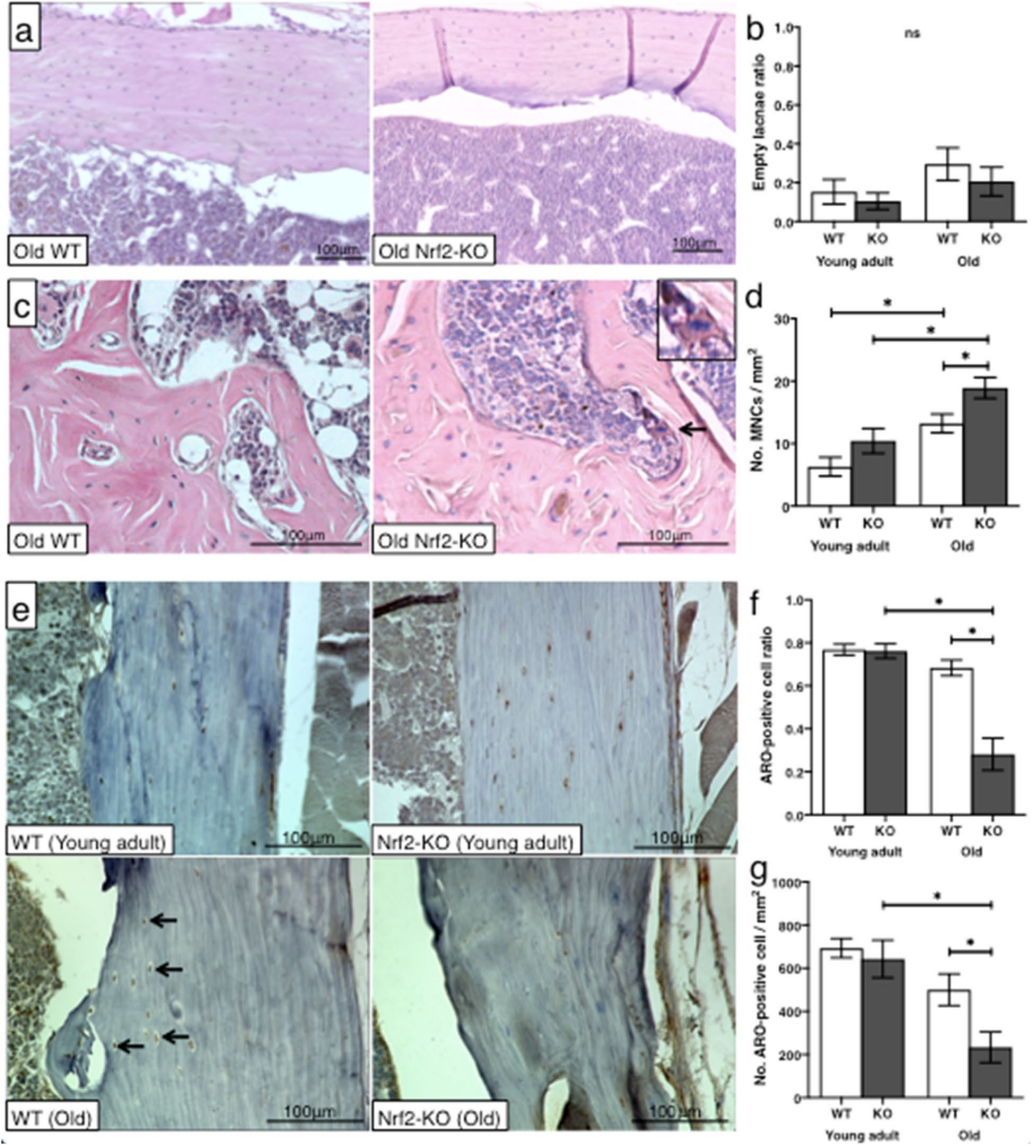
Histological and cytological differences between WT and Nrf2-specific KO bones Comparison of the ratio of empty osteocyte lacunae (**a, b**) and the number of osteoclast-like cells (**c, d**) between WT and Nrf2-specific KO mice at young adult and old age. Representative images of osteocytes in cortical bone lacunae (old WT vs. KO) and osteoclast-like cells in the distal femur (old WT and KO). Representative immunohistochemical staining of aromatase in osteocytes in cortical bone of old WT and KO mice (**e-g**). Aromatase-positive osteocytes are indicated by arrows (**e**). The number of aromatase-positive osteocytes per 1 mm^2^ of cortical bone (**f**) and the ratio (total cell counts) (**g**) between old WT and KO mice. Histology was compared by Wilcoxon rank sum test. Values are means ± SEM. *ρ < 0.05 indicates significance; MNC, multinuclear cell; ARO, aromatase

No difference in empty osteocyte lacunae between young adult and old WT and Nrf2-specific KO mice was found (Fig. 4a, b). Interestingly, old KO mice revealed a higher number of osteoclast-like cells in the cortex as compared old WT mice (p < 0.05). In contrast, there was no significant difference in the numbers between young adult WT and KO mice. Also, the number of osteoclast-like cells in bone of old WT and KO mice was significantly higher than in young adult mice, respectively (*ρ* < 0.05) (Fig. 4c, d). The number of aromatase-positive osteocytes per 1 mm^2^ of bone, and thus the ratio of aromatase-positive cells, in old KO mice were significantly lower than in old WT mice (*ρ* < 0.05). Conversely, there was no difference in aromatase-positive osteocytes between young adult WT and KO mice (Fig. 4e-g).

**Fig. 5:**
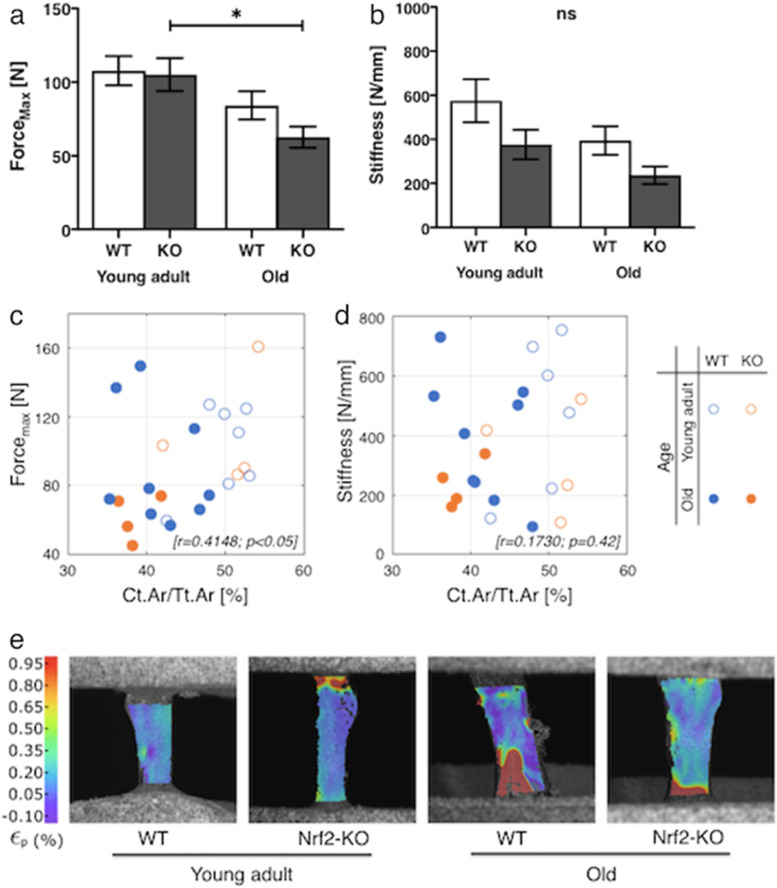
Reduced fracture resilience of old Nrf2-specific KO femurs Peak force [Force_max_ (N)] (**a**) and stiffness (N/mm) (**b**) of femurs from female young adult and old WT mice were compared to Nrf2-specific KO specimens using the Wilcoxon rank sum test. The correlation of force_max_ (N) (**c**) and stiffness (N/mm) (**d**) with cortical bone fraction [Ct.Ar/Tt.Ar (%)] from μCT data was evaluated using the Spearman’s Rank correlation coefficient. Representative full-field strain maps (main principal true strains) of 4 groups of the murine femurs at the failure point by using a Digital Image Correlation (DIC) system. The DIC images are taken at the maximum force values of each specimen (i.e., 150 N, 94.54 N, 41.05 N, and 56.47 N for each specimen from left to right) **(e)**. Values are expressed as means ± SEM. *p < 0.05 indicates significance

The maximum force was significantly lower in femurs from old Nrf2-specific KO mice as compared to bones from young adult KO mice (p < 0.05), whereas no significant difference was detected between young adult and old WT mice. Likewise, no difference in bone stiffness was found between young adult and old WT and KO mice (Fig. 5a, b). The peak force was statistically correlated to cortical μCT parameter Ct.Ar/Tt.Ar (correlation coefficient; r = 0.4148, *ρ* < 0.05), while there was no significant correlation between stiffness and Ct.Ar/Tt.Ar (r = 0.1730, *ρ* = 0.42) (Fig. 5c, d). From DIC measurements, independent of the treatment type, we observed a more homogeneous strain distribution in the femur shaft for young adult groups while, areas with higher strain concentrations were observed for bones at older age (Fig. 5e).

**Fig. 6:**
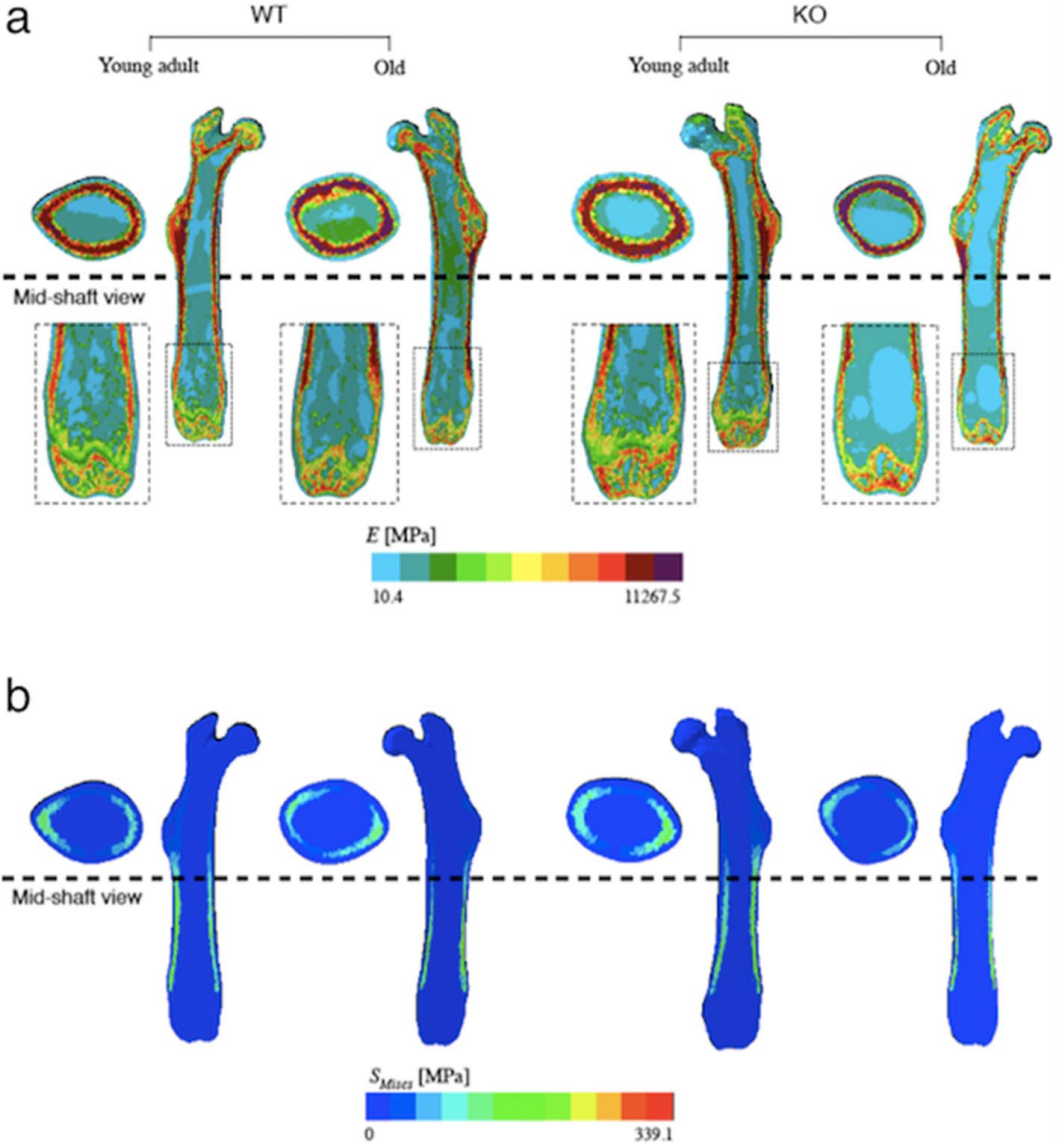
Finite element simulation on selected specimens from young adult WT and Nrf2-specific KO mice, and old WT and KO mice. Material assignment (**a**), and von Mises stress distribution of selected representative bone specimens (**b**)

In the representative images, narrow red zone in the mid cortical and low-grade area in femoral metaphysis based on Young’s modulus were seen in old Nrf2-specific KO mice (Fig. 6a). The maximum von Mises stress was located at the mid-cortical bone in both young adult WT and KO mice. On the other hand, the values of von Mises stress showed less concentrations in those regions in old KO mice (Fig. 6b).

## Discussion

We investigated the effects of systemic Nrf2 deficiency on bone structure and tissue biomechanics in aging females using a murine Nrf2-specific KO model and showed severely compromised trabecular content and BMD in the femoral neck and distal metaphysis of 90-week-old KO mice compared to age- and gender-matched WT littermates. Previous studies also described skeletal phenotypes in female Nrf2-specific KO mice [8, 26, 27], such as decreased BMD in the distal femoral metaphysis of younger, 6–9-month-old, female C57BL/J6 Nrf2-KO mice [26]. In contrast, no differences in trabecular BV/TV or Tb. Th of lumber vertebrae or femoral BMD in 15-month-old Nrf2-specific KO animals were found by dual-energy X-ray absorptiometry (DEXA) [8]. Of note, all mice in previous studies were much younger than in our study and discrepancies between the results could be due to age. Moreover, earlier studies did not disclose the selected anatomical region of the bones precisely enough to allow a direct comparison as this can strongly influence the results [18]. We analyzed the femur as the longest limb bone and established an accurate and reproducible way to evaluate relevant bone parameters in accordance with previous reports [14, 15, 17–19]. No differences in Tb. Th, trabecular BMD, and BV/TV between 12-week-old young adult WT and Nrf2-specific KO mice could be found in our study, which is consistent with Kim et al. [27] using 3 month-old female C57BL/J6 Nrf2-specific KO mice and Pellegrini et al. [8] using 3-month-old female mice. In our study, complete loss of trabeculae in the distal femoral metaphyseal region was seen in 60% of all old Nrf2-specific KO animals, despite presence of abundant trabeculae age- and gender-matched WT littermates. Therefore, our results suggest that chronic Nrf2 deficiency for 90 weeks decreases the quantity of trabeculae and contributes to osteoporotic change in elderly females. Our results further show that commonly analyzed cortical bone parameters are also reduced in old Nrf2-specific KO mice compared to age-matched WT mice, despite absence of such differences between younger animals. To our best of knowledge, no previous studies report such findings on cortical femoral bone parameters of in Nrf2-specific KO mice, but a report on decreased cortical bone area in lumber vertebrae of 6–9-month-old female Nrf2-specific KO mice is in line with our data [26].

Mechanical loading and DIC-coupled FE modeling further identified increased bone fragility in old Nrf2-specific KO mice, but no difference in their stiffness values. Despite differences in age and gender, this is roughly in line with the only other report performing mechanical testing on murine Nrf2-specific KO bones [28], reporting reduced force and energy to failure in 16-week-old male animals. Interestingly, also these authors did not find difference in stiffness between Nrf2-specific KO and WT mice. Our mechanical tests indicate that the loss of bone quality in old Nrf2-specific KO mice correlates with a decreased bone quantity. Our mechanical tests were carried out by fixing both ends of the femur in order to focus on the diaphysis. On the other hand, we saw major changes in the bone quality of the femoral neck region on μCT analysis as well as diaphysis. However, due to the small size of the mouse femur, it was quite challenging to carry out controlled mechanical tests only on the femoral neck.

We could not find a difference in empty osteocyte lacunae, an indicator of osteoporosis or reduced bone remodeling [29, 30], between WT and Nrf2-specific KO mice. In contrast, a significantly higher number of osteoclast-like cells was found in old Nrf2-KO mice compared to old WT controls, which is in agreement with a report of increased osteoclast activity in Nrf2-specific KO vertebrae compared to WT mice [26]. With regard to osteoclastogenesis, in murine osteoclast progenitor cells, receptor activator of NF-kappaB ligand (RANKL) stimulation increased Kelch-like erythroid cell-derived protein with cap ‘n’ collar homology-associated protein 1 (Keap1) (Keap1; Nrf2 antagonist), which is an Nrf2 antagonist [31]. Thus, consequently decreasing Nrf2 resulted in a decreased Nrf2/Keap1 ratio and reduced cytoprotection. In contrast, overexpression of Nrf2 increased the expression of cytoprotective enzymes, reduced reactive oxygen species (ROS) levels, and decreased the number of tartrate-resistant acid phosphatase (TRAP)-positive multinuclear cells and inhibited osteoclast differentiation and bone resorption [31]. As osteoporosis results from an imbalance in bone turnover in favor of osteoclast activity, our results suggest that increased osteoclast-like cell numbers cause osteoporotic progression in old Nrf2 KO animals.

In a physiological state, Keap1 suppresses Nrf2 activity [32]. And, 3-month-old male heterozygotic Keap1 mice had significantly higher BV/TV and lower osteoclast number compared to age- and gender-matched WT controls [33]. This suggests that moderate Nrf2 activation through Keap1 disruption can improve bone mass and agrees with our data. Additionally, a lower bone mineral density in 10–12 week male Keap1-specific KO mice is in agreement with this.

Expression of aromatase, also known as estrogen synthase (EC 1.14.14.1, CYP19A1), catalyzing the last steps of estrogen biosynthesis from circulating androgens [34], was significantly reduced in cortical osteocytes from our old Nrf2-specific KO mice. Expression of aromatase in human cortical osteocytes and osteoblasts was reported earlier [35]. Estrogen deficiency is causing primary osteoporosis in postmenopausal females [12], and 17β-estradiol (E2) was shown to induce the Nrf2-Keap1 antioxidant defense system in other tissues [9–11]. Although the direct relationship between Nrf2 and the aromatase-estrogen pathway in bone is not yet clear, previous reports suggest the importance of Nrf2 under estrogen-deficient conditions in this tissue, too [26, 36]. Reduced bone parameters and increased osteoclast activity were reported from 6 to 9-month-old ovariectomized Nrf2-specific KO mice [26] and higher oxidative stress was observed in these mice. If epigenetic de-repression of Nrf2 mediates these osteoprotective effects as shown for running exercise in ovariectomized animals [36] has to be shown. Such reports are in line with our findings and cumulatively strongly suggest that disturbed estrogen metabolism might have contributed to the osteoporotic phenotype of old Nrf2-specific KO mice. Moreover, in relation to estrogen, menstruation is reported to occur in 80% of female mice with irregular cycles by 17 months of age, and all females are acyclic by 25 months. The age of the mice we investigated, 90 weeks, is within this range and this is a unique aspect distinguishing our study from all others in Nrf2-specific KO study [37]. Thus, during the first 6 months of life, estrogen levels were critically low in our old mice, which may have affected the Nrf2 system specifically.

Interestingly, ROS are known to adversely affect bone homeostasis and fracture healing [5, 38]. As Nrf2 protects cells against ROS exposure [39] it may not surprise that the expression of Nrf2-target genes is decreased in female KO mice [8]. As Nrf2 plays an important role in maintaining redox homeostasis, Nrf2 deficiency would result in ROS exposure. Certain ROS levels are described to suppress osteoblastic function [40] and MSC differentiation [41], which are both important for bone formation. Another study suggested that ROS can activate osteoclast precursors and promote bone resorption [42], which was later confirmed by showing that Nrf2 loss can directly promote osteoclast function [31]. However, none of these studies looked at the long-term response as we did. Thus, a life-long Nrf2 deficiency is likely to result in bone aging (= osteoporosis) through loss of antioxidant activity. We were the first to show that Nrf2 expression is activated during bone fracture healing and that bone healing and remodeling in Nrf2-specific KO animals was retarded as compared to the WT mice [7], resulting in an immature bone callus. This might have been due to a reduced osteoclastic activity, which usually precedes anabolic bone repair by osteoblasts. Considering that osteoporotic fractures are still challenging for orthopaedic surgeons, pharmacological Nrf2 induction may become a valuable future therapeutic strategy for these patients.

Intriguingly, intrinsic oxidative stress condition such as type-2 diabetes mellitus, or heavy smoking, inhibits bone regeneration and, consequently impairs fracture healing [5]. Clinically, osteoporotic postmenopausal women are known for lower levels of antioxidant enzymes [43].

A limitation of our study is that we only investigated bone quantity at two different ages in females. However, together with previous reports on likewise altered bone parameters in Nrf2-specific KO mice of different ages [8, 26, 27], we conclude that effects of Nrf2 deficiency on bone quality may vary with age or gender. We believe that our 90-week-old murine model provides important information about the change in bone quantity in elderly females with chronic Nrf2 deficiency. In continuation of the reported data, longitudinal studies, also including detailed comparisons with vertebrae from age- and gender-matched Nrf2-specific KO mice are currently in preparation. Furthermore, the mechanism of involvement of Nrf2 in osteoporosis has still not been fully elucidated, yet. Future studies should validate Nrf2 expression levels in osteoporotic animal models or in biological samples from osteoporotic patients. Additionally, the intersection of ROS signaling and the NF-kappaB-mediated inflammatory signaling pathways should be studied in Nrf2 deficient osteoporotic models in the future to explore the relationship between Nrf2 loss and osteoporosis in more detail.

## Conclusion

In our study, old Nrf2-specific KO mice showed reduced trabecular and cortical bone quantity and strongly suggests that chronic Nrf2 deficiency for up to 90 weeks contributes to the progression of osteoporosis in females. In particular, complete loss of trabecular bone in the distal femoral metaphysis in 60% of all old Nrf2-specific KO mice is indicative of severe osteoporosis. This is in line with a decreased peak force to induce bone fracture in these old KO animals. Furthermore, reduced estrogen synthase (i.e., aromatase) expression in old Nrf2-specific KO mice provides circumstantial evidence for a potentially severely imbalanced bone due to Nrf2 deficiency, resulting in low bone mass and fragility.

## Data Availability

The datasets used and/or analyzed during the current study available from the corresponding author on reasonable request.

## Abbreviations

BMD: bone mineral density
BS: bone surface
BV/TV: bone volume fraction
Ct.Ar: cortical bone area
Ct.Ar/Tt.Ar: cortical bone fraction
Ct.Th: cortical thickness
DIC: Digital Image Correlation
DEXA: dual-energy X-ray absorptiometry
EDTA: ethylenediaminetetraacetic acid
E2: estradiol
FE: finite element
HE: hematoxylin and eosin
HU: Hounsfield Unit
Keap1: Kelch-like erythroid cell-derived protein with cap ‘n’ collar homology-associated protein 1
KO: knockout
μCT: Micro computed tomography
Nrf2: nuclear factor erythroid 2–related factor 2
PBS: phosphate buffer saline
ROS: reactive oxygen species
RANKL: receptor activator of NF-kappaB ligand
ROI: region of interest
SEM: standard error of the mean
TRAP: tartrate-resistant acid phosphatase
Tt.Ar: total cross-sectional area
Tb.Th: trabecular thickness
WT: wild-type

## References

[1] Sies H (2020). Oxidative stress: concept and some practical aspects. Antioxidants (Basel)..

[2] Kimball JS, Johnson JP, Carlson DA (2021). Oxidative stress and osteoporosis. J Bone Joint Surg Am.

[3] Schröder K (2019). NADPH oxidases in bone homeostasis and osteoporosis. Free Radic Biol Med.

[4] Huang X, Shu H, Ren C, Zhu J (2022). SIRT3 improves bone regeneration and rescues diabetic fracture healing by regulating oxidative stress. Biochem Biophys Res Commun.

[5] Kubo Y, Wruck CJ, Fragoulis A, Drescher W, Pape HC, Lichte P, Fischer H, Tohidnezhad M, Hildebrand F, Pufe T, Jahr H (2019). Role of Nrf2 in fracture healing: clinical aspects of oxidative stress. Calcif Tissue Int.

[6] Bellezza I, Giambanco I, Minelli A, Donato R (2018). Nrf2-Keap1 signaling in oxidative and reductive stress. Biochim Biophys Acta Mol Cell Res.

[7] Lippross S, Beckmann R, Streubesand N, Ayub F, Tohidnezhad M, Campbell G, Kan YW, Horst F, Sönmez TT, Varoga D, Lichte P, Jahr H, Pufe T, Wruck CJ (2014). Nrf2 deficiency impairs fracture healing in mice. Calcif Tissue Int.

[8] Pellegrini GG, Cregor M, McAndrews K, Morales CC, McCabe LD, McCabe GP, Peacock M, Burr D, Weaver C, Bellido T (2017). Nrf2 regulates mass accrual and the antioxidant endogenous response in bone differently depending on the sex and age. PLoS One.

[9] Chen CS, Tseng YT, Hsu YY, Lo YC (2013). Nrf2-Keap1 antioxidant defense and cell survival signaling are upregulated by 17β-estradiol in homocysteine-treated dopaminergic SH-SY5Y cells. Neuroendocrinology..

[10] Yu J, Zhao Y, Li B, Sun L, Huo H (2012). 17β-estradiol regulates the expression of antioxidant enzymes in myocardial cells by increasing Nrf2 translocation. J Biochem Mol Toxicol.

[11] Wu J, Williams D, Walter GA, Thompson WE, Sidell N (2014). Estrogen increases Nrf2 activity through activation of the PI3K pathway in MCF-7 breast cancer cells. Exp Cell Res.

[12] Cheng CH, Chen LR, Chen KH (2022). Osteoporosis due to hormone imbalance: an overview of the effects of estrogen deficiency and glucocorticoid overuse on bone turnover. Int J Mol Sci.

[13] Fragoulis A, Schenkel J, Herzog M, Schellenberg T, Jahr H, Pufe T, Trautwein C, Kensler TW, Streetz KL, Wruck CJ (2019). Nrf2 ameliorates DDC-induced Sclerosing cholangitis and biliary fibrosis and improves the regenerative capacity of the liver. Toxicol Sci.

[14] Bouxsein ML, Boyd SK, Christiansen BA, Guldberg RE, Jepsen KJ, Müller R (2010). Guidelines for assessment of bone microstructure in rodents using micro-computed tomography. J Bone Miner Res.

[15] Kohler T, Beyeler M, Webster D, Muller R. Compartmental bone morphometry in the mouse femur: repr1oducibility and resolution dependence of microtomographic measurements. Calcif Tissue Int. 2005;77:281–90. 10.1007/s00223-005-0039-2. 10.1007/s00223-005-0039-2 16283571

[16] Gremse F, Stärk M, Ehling J, Menzel JR, Lammers T, Kiessling F (2016). Imalytics preclinical: interactive analysis of biomedical volume data. Theranostics..

[17] Brommage R, Jeter-Jones S, Xiong W, Liu J (2021). MicroCT analyses of mouse femoral neck architecture. Bone..

[18] Campbell GM, Sophocleous A (2014). Quantitative analysis of bone and soft tissue by micro-computed tomography: applications to ex vivo and in vivo studies. Bonekey Rep.

[19] Marenzana M, Greenslade K, Eddleston A, Okoye R, Marshall D, Moore A, et al. Sclerostin antibody treatment enhances bone strength but does not prevent growth retardation in young mice treated with dexamethasone. Arthritis Rheum. 2011;63:2385–95. 10.1002/art.30385. 10.1002/art.30385 21484764

[20] Buie HR, Campbell GM, Klinck RJ, MacNeil JA, Boyd SK. Automatic segmentation of cortical and trabecular compartments based on a dual threshold technique for in vivo micro-CT bone analysis. Bone. 2007;41:505–15. 10.1016/j.bone.2007.07.007. 10.1016/j.bone.2007.07.007 17693147

[21] Hamann C, Rauner M, Ho’hna Y, Bernhardt R, Mettelsiefen J, Goettsch C, et al. Sclerostin antibody treatment improves bone mass, bone strength, and bone defect regeneration in rats with type 2 diabetes mellitus. J Bone Miner Res. 2013;28:627–38. 10.1002/jbmr.1803. 10.1002/jbmr.1803 23109114

[22] Parkinson IH, Badiei A, Fazzalari NL. Variation in segmentation of bone from micro-CT imaging: implications for quantitative morphometric analysis. Australas Phys Eng Sci Med. 2008;31:160–4. 10.1007/BF03178592. 10.1007/BF03178592 18697709

[23] Brooks PJ, Glogauer M, McCulloch CA (2019). An overview of the derivation and function of multinucleated Giant cells and their role in pathologic processes. Am J Pathol.

[24] Kubo Y, Motomura G, Ikemura S, Hatanaka H, Fukushi JI, Hamai S, Yamamoto T, Nakashima Y (2018). Osteoclast-related markers in the hip joint fluid with subchondral insufficiency fracture of the femoral head. J Orthop Res.

[25] Ciarelli MJ, Goldstein SA, Kuhn JL, Cody DD, Brown MB (1991). Evaluation of orthogonal mechanical properties and density of human trabecular bone from the major metaphyseal regions with materials testing and computed tomography. J Orthop Res.

[26] Ibanez L, Ferrandiz ML, Brines R, Guede D, Cuadrado A, Alcaraz MJ (2014). Effects of Nrf2 deficiency on bone microarchitecture in an experimental model of osteoporosis. Oxidative Med Cell Longev.

[27] Kim JH, Singhal V, Biswal S, Thimmulappa RK, Digirolamo DJ (2014). Nrf2 is required for normal postnatal bone acquisition in mice. Bone Res.

[28] Sun YX, Li L, Corry KA, Zhang P, Yang Y, Himes E, Mihuti CL, Nelson C, Dai G, Li J (2015). Deletion of Nrf2 reduces skeletal mechanical properties and decreases load-driven bone formation. Bone..

[29] Milovanovic P, Busse B (2020). Phenomenon of osteocyte lacunar mineralization: indicator of former osteocyte death and a novel marker of impaired bone quality?. Endocr Connect.

[30] Bonewald LF (2011). The amazing osteocyte. J Bone Miner Res.

[31] Kanzaki H, Shinohara F, Kajiya M, Kodama T. The Keap1/Nrf2 protein axis plays a role in osteoclast differentiation by regulating intracellular reactive oxygen species signaling. J Biol Chem. 2013;288:23009–20. 10.1074/jbc.M113.478545. 10.1074/jbc.M113.478545 PMC3743476 23801334

[32] Horie Y, Suzuki T, Inoue J, Iso T, Wells G, Moore TW, Mizushima T, Dinkova-Kostova AT, Kasai T, Kamei T, Koshiba S, Yamamoto M (2021). Molecular basis for the disruption of Keap1-Nrf2 interaction via Hinge & Latch mechanism. Commun Biol.

[33] Yin Y, Corry KA, Loughran JP, Li J (2020). Moderate Nrf2 activation by genetic disruption of Keap1 has sex-specific effects on bone mass in mice. Sci Rep.

[34] Merlotti D, Gennari L, Stolakis K, Nuti R (2011). Aromatase activity and bone loss in men. J Osteoporos.

[35] Sasano H, Uzuki M, Sawai T, Nagura H, Matsunaga G, Kashimoto O, Harada N (1997). Aromatase in human bone tissue. J Bone Miner Res.

[36] Chen X, Zhu X, Wei A, Chen F, Gao Q, Lu K, Jiang Q, Cao W (2021). Nrf2 epigenetic derepression induced by running exercise protects against osteoporosis. Bone Res.

[37] Frick KM, Burlingame LA, Arters JA, Berger-Sweeney J. Reference memory, anxiety and estrous cyclicity in C57BL/6NIA mice are affected by age and sex. Neuroscience. 2000;95:293–307. 10.1016/s0306-4522(99)00418-2. 10.1016/s0306-4522(99)00418-2 10619486

[38] Bădilă AE, Rădulescu DM, Ilie A, Niculescu AG, Grumezescu AM, Rădulescu AR (2022). Bone regeneration and oxidative stress: an updated overview. Antioxidants (Basel).

[39] Al-Sawaf O, Clarner T, Fragoulis A, Kan YW, Pufe T, Streetz K, Wruck CJ (2015). Nrf2 in health and disease: current and future clinical implications. Clin Sci (Lond).

[40] Arai M, Shibata Y, Pugdee K, Abiko Y, Ogata Y. Effects of reactive oxygen species (ROS) on antioxidant system and osteoblastic differentiation in MC3T3-E1 cells. IUBMB Life. 2007;59:27–33. 10.1080/15216540601156188. 10.1080/15216540601156188 17365177

[41] Vanella L, Sanford C Jr, Kim DH, Abraham NG, Ebraheim N. Oxidative stress and heme oxygenase-1 regulated human mesenchymal stem cells differentiation. Int J Hypertens. 2012;2012:890671. 10.1155/2012/890671. 10.1155/2012/890671 PMC3296285 22518296

[42] Yamasaki N, Tsuboi H, Hirao M, Nampei A, Yoshikawa H, Hashimoto J. High oxygen tension prolongs the survival of osteoclast precursors via macrophage colony-stimulating factor. Bone. 2009;44:71–9. 10.1016/j.bone.2008.09.015. 10.1016/j.bone.2008.09.015 18973838

[43] Maggio D, Barabani M, Pierandrei M, Polidori MC, Catani M, Mecocci P, Senin U, Pacifici R, Cherubini A (2003). Marked decrease in plasma antioxidants in aged osteoporotic women: results of a cross-sectional study. J Clin Endocrinol Metab.

